# Applied anatomy of the skull in the Arabian horse: A computed tomographic, cross‐sectional, volumetric and morphometric study

**DOI:** 10.1002/vms3.618

**Published:** 2021-08-27

**Authors:** Nader Goodarzi, Omid Zehtabvar, Mohsen Tohidifar

**Affiliations:** ^1^ Faculty of Veterinary Medicine Department of Basic Sciences and Pathobiology Razi University Kermanshah Iran; ^2^ Faculty of Veterinary Medicine Department of Basic Sciences University of Tehran Tehran Iran; ^3^ Faculty of Veterinary Medicine Razi University Kermanshah Iran

**Keywords:** anaesthesia, Arabian horse, paranasal sinus, skull, volume

## Abstract

This study was conducted to present a comprehensive and integrative computed tomography (CT) – anatomical cross sections atlas of skull, volumetric properties of the paranasal sinuses, and morphometric values for surface cranial nerves in the adult Arabian horse. Ten heads of Arabian horse breed were used. The different structures in the nasal, oral and cranial cavities were determined and labelled in the anatomical sections and their corresponding CT scan images. Three paranasal sinuses namely maxillary, conchofrontal and sphenopalatine sinuses were identified in the CT scan images. The caudal maxillary sinus was the largest paranasal sinus with 131.93 ± 7.67 cm^3^ volume and the sphenopalatine sinus 13.3 ± 1.2 cm^3^ volume was the smallest one. The infraorbital foramen was located 4.16 ± 0.18 cm and 4.70 ± 0.35 cm far away from the most rostral point of the facial crest and alveolar root, respectively. The mean distance between the mental foramen and most lateral incisive tooth was 3.12 ± 0.29 cm. These results including present CT scan‐cross‐sectional atlas, paranasal sinuses volume and morphometric properties would be applicable in practice for more precise diagnosis of head lesions and blocking the surface terminal branches of the cranial nerves during surgical operations in this valuable horse's breed.

## Graphical abstract

Three paranasal sinuses namely maxillary, conchofrontal and sphenopalatine sinuses in the Arabian horse. The caudal maxillary sinus was the largest paranasal sinus and the sphenopalatine sinus was the smallest one in the Arabian horse

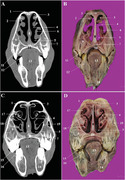

## 1 INTRODUCTION

The interpretation of the equine skull images and determining the relationship between its different structures is challenging for students and practitioners (Morrow et al., 2000). Imaging techniques are considerably helpful for solving this issue. However, each modalities has its own application according to the diagnostic needs. In comparison to traditional radiography, CT scan imaging provides more detailed appearance of head structures in cross section. The most remarkable weakness of radiography is the superimposition of bony structures and cavities that made it difficult to distinguish them from each other perfectly (Solano & Brawer, 2004). In addition, CT scan images can be manipulated by user to achieve the best contrast between adjacent soft and bony tissues (Frazho et al., 2008; Losonsky et al., 1997). Undoubtedly, a thorough theoretical working knowledge of the skull anatomy is needed before interpreting the CT scan images of the skull.

In veterinary practice, usage of the CT scan procedure for investigating the anatomy of the skull regions in different mammals (Arencibia et al., 2000; De Rycke et al., 2003; El‐Gendy & Alsafy, 2010; Frazho et al., 2008; Reetz et al., 2006; Saunders et al., 2012; Smallwood et el., 2002; Zotti et al., 2009) and non‐mammals species (Banzato et al., 2010) has a long history. This modality has previously been used for studying the CT scan appearance of the skull in horse (Arencibia et al., 2000 ; Morrow et al., 2000), camel (Alsafy et al., 2014), Egyptian buffalo (Alsafy et al., 2013) and donkey (El‐Gendy & Alsafy, 2010, El‐Gendy et al., 2014).

In the present study, we attempted to provide some applied information about the skull anatomy of the adult Arabian horse for the first time. At first, a CT cross‐sectional atlas of the head was presented for better understanding the normal appearance of the head structures in CT scan images. Second, the normal volume of the paranasal sinuses were estimated using design‐based unbiased stereological procedures, and finally, morphometric properties related to the superficial cranial nerves were measured for using in local anaesthesia and nerves blocking before surgical operations or tooth extraction.

## 2 MATERIALS AND METHODS

Ten heads of adult Arabian horse were used. The head samples were obtained from animals who were euthanized due to diseases unrelated to the head injuries, dental diseases or sinusal diseases. All the horses had their own history and certificates. They had the same diet and training management and also the same farrier. The heads were disarticulated from atlantooccipital joint and were used immediately for CT scan imaging. After imaging, the samples were freezed in –20°C temperature for anatomical cross‐section preparation.

### 2.1 Computed tomographic imaging

The fresh head samples were immediately (up to 2 h after death) examined using a helical scanner (Siemens Somatom®, 2 detectors, Germany/Kvp: 130 V, mAs: 72 and slice thickness: 2 mm, WW: 1500 and WL:,450). All CT images in bone and soft tissue reconstruction algorithm were retrieved and reviewed using an image analysis workstation (Clear Canvas by Synaptive Medical, Toronto, Canada). The CT images were coupled with their corresponding anatomical cross‐sections and all anatomical structures were labelled carefully. Then, 3D reconstruction was done using the using RadiAnt DICOM Viewer software.

### 2.2 Cross‐section preparation

After 1 week, the frozen samples were serially sectioned using an electric band saw. Ten transverse sections with about 3 cm thickness were harvested from each sample. The caudal aspect of the sections was cleaned gently using a light brush under tap water. After drying in room temperature, the caudal surface of the sections were photographed using a digital camera (Canon, SX130‐IS, 12 MP, Japan). The images were processed and anatomical structures were identified and labelled using Adobe Photoshop CC (Adobe system, San Jose, CA, USA).

### 2.3 Volumetric study

**FIGURE 1:**
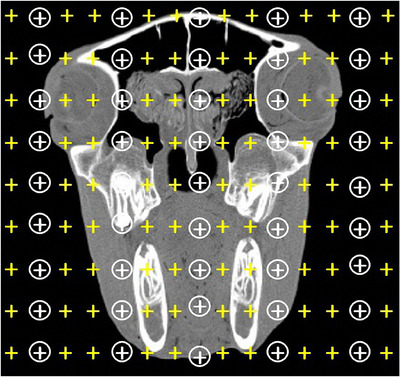
Cavalieri principle for estimating the fractional volume of paranasal sinuses. A compound point grid was lied on the obtained CT image

Cavalieri principle was used for estimating the volume of desired structures. A point grid composed of 80 crosses (+) was superimposed on the consecutive CT images (197 images per sample) and the total volume of the head was estimated using the following formula (Mass et al., 2009; Losonsky et al., 1997) (Figure 1):

(1) $$V_{total}=\sum P\times {\left[SU\times d/SL\right]}^{2}\times t,$$

*∑P* = total points hitting the whole sections, *SU = *scale unit, *d* = distance between two points, *SL* = scale length, *t* = section interval.

The volume density of paranasal sinuses was determined using the following formula (Howard & Reed, 2005):

(2) $${V_{v}}_{\left(structure/reference\right)}=\sum P_{structure}/\sum P_{reference},$$

*∑P_structure_*_ _=_ _total points hitting the desire structures, *∑P_reference_*_ _= total points hitting the reference volume. The values of volume densities were converted to the total volume by multiplying the volume density by the reference volume.

### 2.4 Morphometric measurements

**FIGURE 2:**
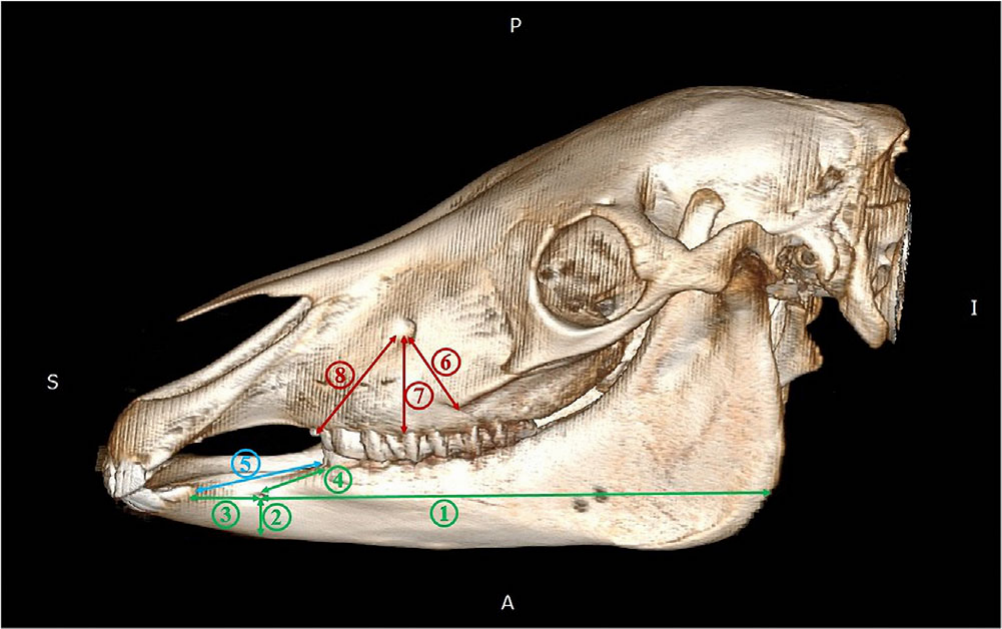
Lateral views of 3D reconstruction of the skull and mandible in the Arabian horse used for measuring morphometric parameters. 1: Mental foramen to the caudal border of the mandibular ramus (MFCB), 2: mental foramen to the ventral border of the mandibular body (MFVB), 3: mental foramen to the most lateral incisive tooth (MFIT), 4: mental foramen to the first premolar tooth (MFPT), 5: bar length (BL), 6: the distance between the infraorbital foramen and facial crest (IF), 7: the distance between the infraorbital foramen to alveolar tooth (IA), 8: the distance between the infraorbital foramen and first upper premolar tooth (IP)

The 3D images underwent morphometric measurements in RadiAnt DICOM Viewer software and eight parameters related to the surface cranial nerves were measured as below (Figure 2):

1. BL (Bar Length): length was the distance between the most lateral incisive tooth and first premolar teeth.
2. MFCB (Mental Foramen to the Caudal Border): it was measured as a distance from the mental foramen to the caudal border of the mandibular ramus.
3. MFVB (Mental Foramen to the Ventral Border): it was measured as a distance from the mental foramen to the ventral border of the mandibular body.
4. MFIT (Mental Foramen to the Incisive Tooth): it was measured as a distance from the mental foramen to the most lateral incisive tooth.
5. MFPT (Mental Foramen to the Premolar Tooth): it was measured as a distance from the mental foramen to the cranial border of the first premolar tooth.
6. IF (Facial crest and Infraorbital foramen): it was the distance between the facial crest and infraorbital foramen.
7. AI (Infraorbital foramen and the Alveolar root): it was the distance between the infraorbital foramen and the alveolar root.
8. IP (Infraorbital foramen and first upper Premolar tooth): it was the distance between the infraorbital foramen and first upper premolar tooth.

## 3 RESULTS

### 3.1 Computed tomographic and cross‐sectional anatomy

**FIGURE 3:**
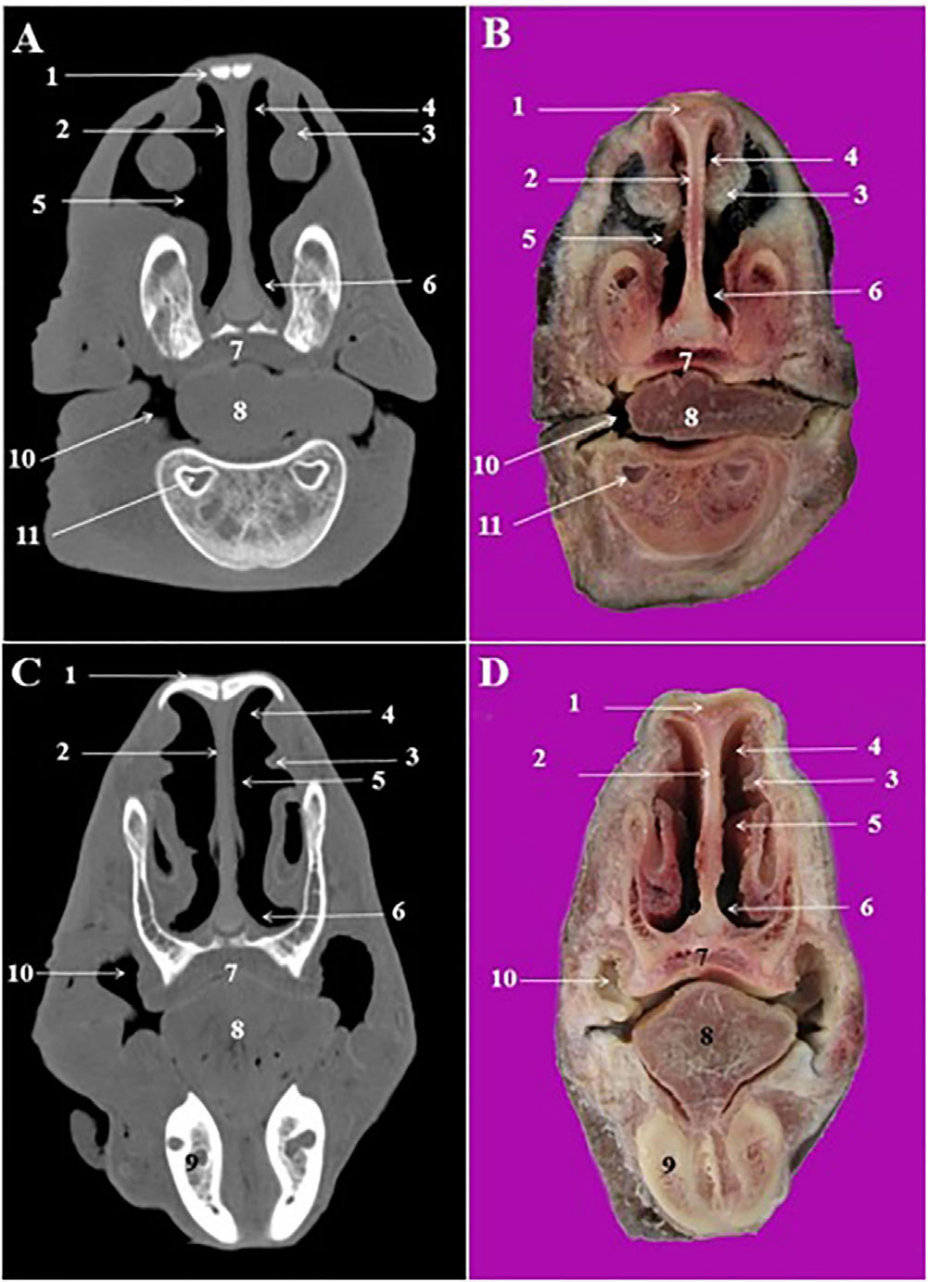
CT scan (a, c) and cross‐sectional (b, d) images of the rostral nasal cavity at the level of the interalveolar space in the Arabian horse. 1: Nasal bone, 2: nasal septum, 3: straight fold of dorsal nasal concha, 4: dorsal nasal meatus, 5: middle nasal meatus, 6: ventral nasal meatus, 7: hard palate, 8: tongue, 9: interalveolar space, 10: buccal vestibule, 11: marrow cavity of mandible

**FIGURE 4:**
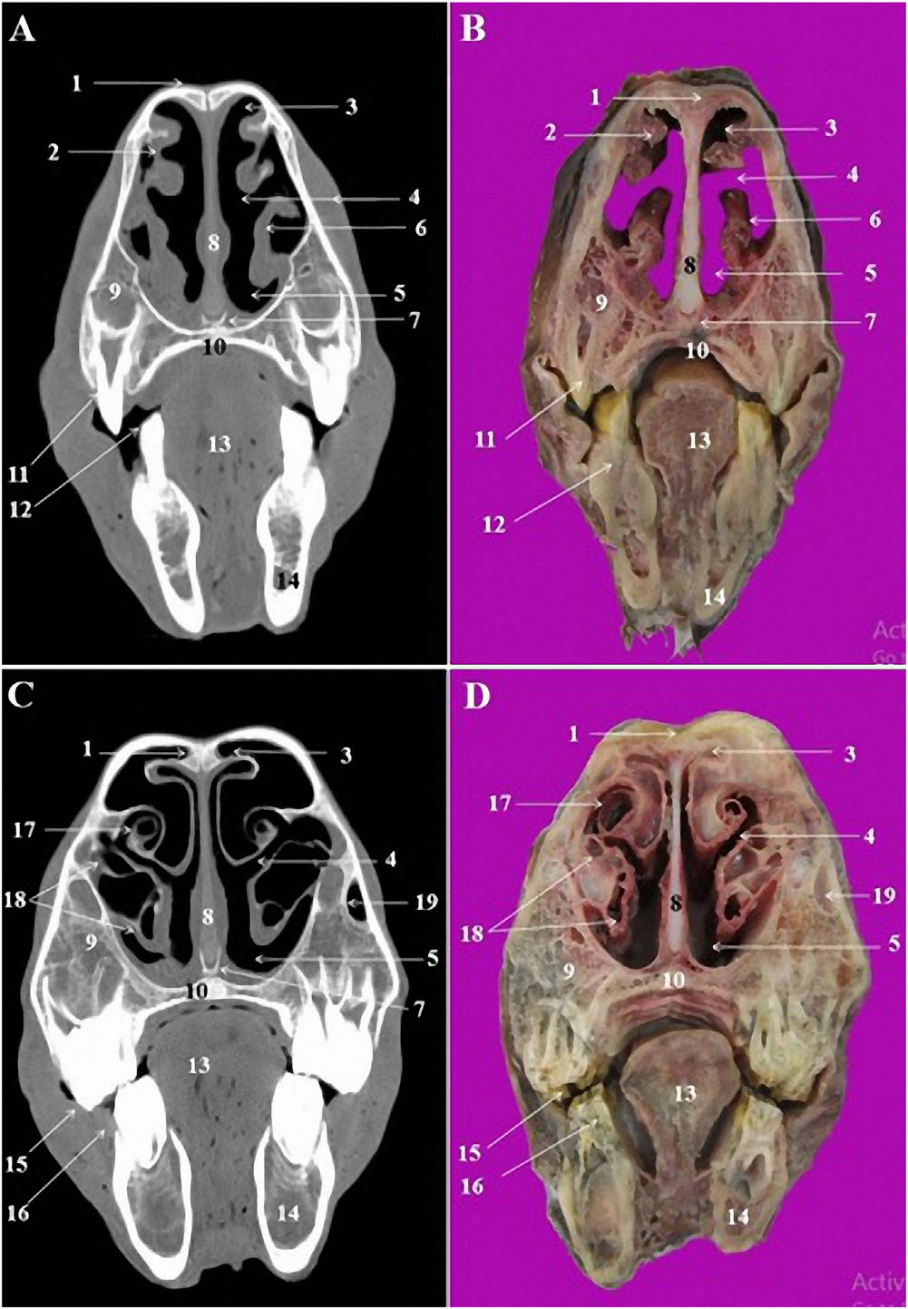
CT scan (a, c) and cross‐sectional (b, d) images of the middle nasal cavity at the level of the first and second premolar teeth in the Arabian horse. 1: Nasal bone, 2: dorsal nasal concha, 3: dorsal nasal meatus, 4: middle nasal meatus, 5: ventral nasal meatus, 6: alar fold of ventral nasal concha, 7: vomer bone, 8: nasal septum, 9: maxillary bone, 10: hard palate, 11: 2nd upper premolar tooth (Triadan 106 and 206), 12: 1th lower premolar tooth (Triadan 305 and 405), 13: tongue, 14: body of mandible, 15: 3rd upper premolar tooth (Triadan 107 and 207), 16: 2nd lower premolar tooth (Triadan 306 and 406), 17: dorsal nasal concha, 18: dorsal and ventral spiral lamellae of the ventral nasal concha, 19: nasolacrimal duct

**FIGURE 5:**
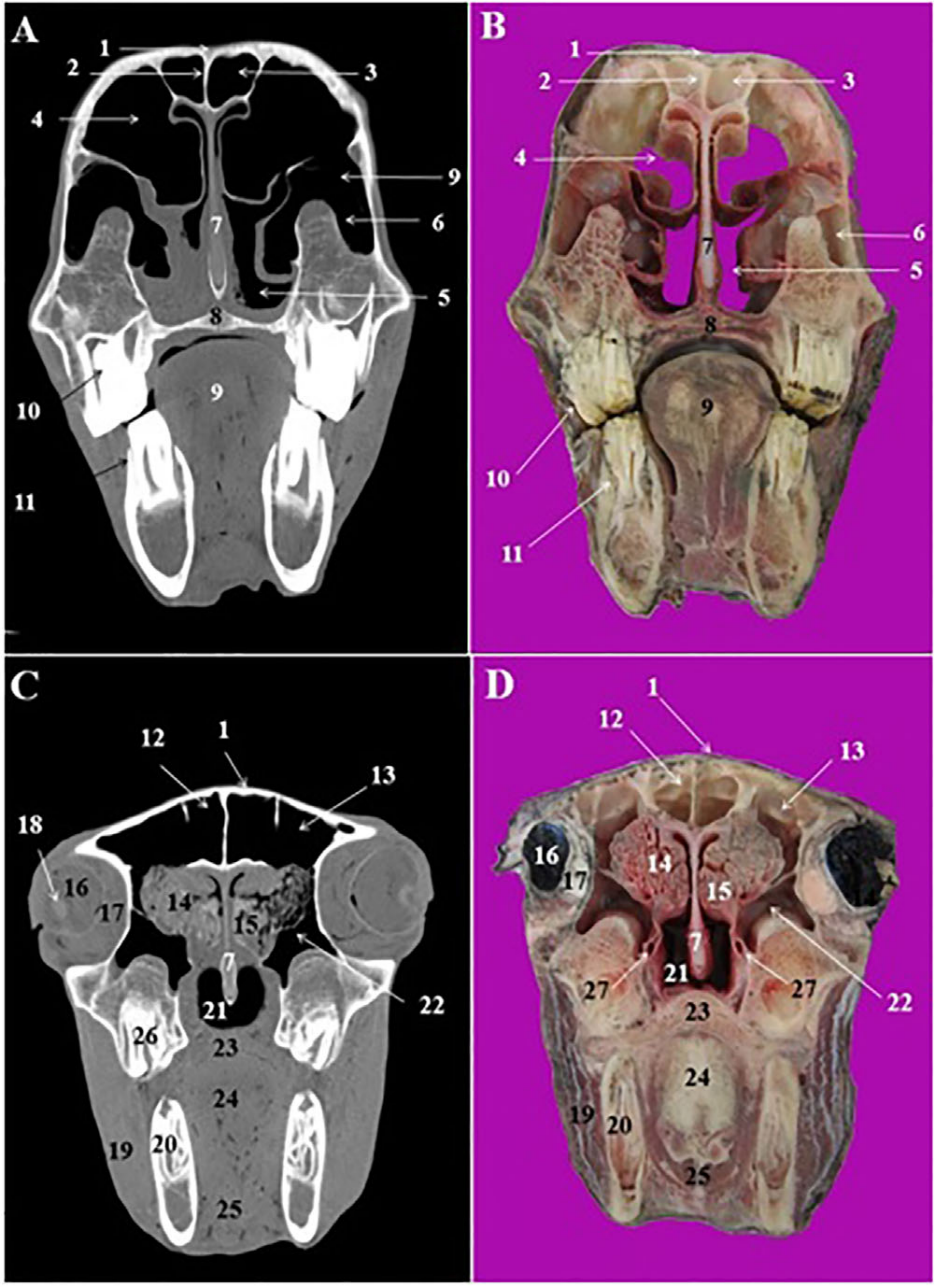
CT scan (a, c) and cross‐sectional (b, d) images of the middle and caudal nasal cavity at the level of the orbit in the Arabian horse. 1: Nasal bone, 2: frontal septum between right and left frontal sinus, 3: rostral frontal sinus, 4 conchofrontal sinus, 5: rostral maxillary sinus, its lateral compartment, 6: medial compartment of rostral maxillary sinus, 7: nasal septum, 8: hard palate, 9: tongue, 10: 1st upper molar tooth (Triadan 109 and 209), 11: 1st lower molar tooth (Triadan 309 and 409), 12: medial frontal sinus, 13: caudal frontal sinus, 14: ethmoidal labyrinth, 15: middle nasal concha, 16: eye, 17: periorbital fat and ocular muscles, 18: lens, 19: masseter muscle, 20: 2nd lower molar tooth (Triadan 310 and 410), 21: nasopharynx, 22: caudal maxillary sinus, 23: soft palate, 24: root of the tongue, 25: omohyoid muscle, 26: 3rd upper molar tooth (Triadan 111 and 211)

**FIGURE 6:**
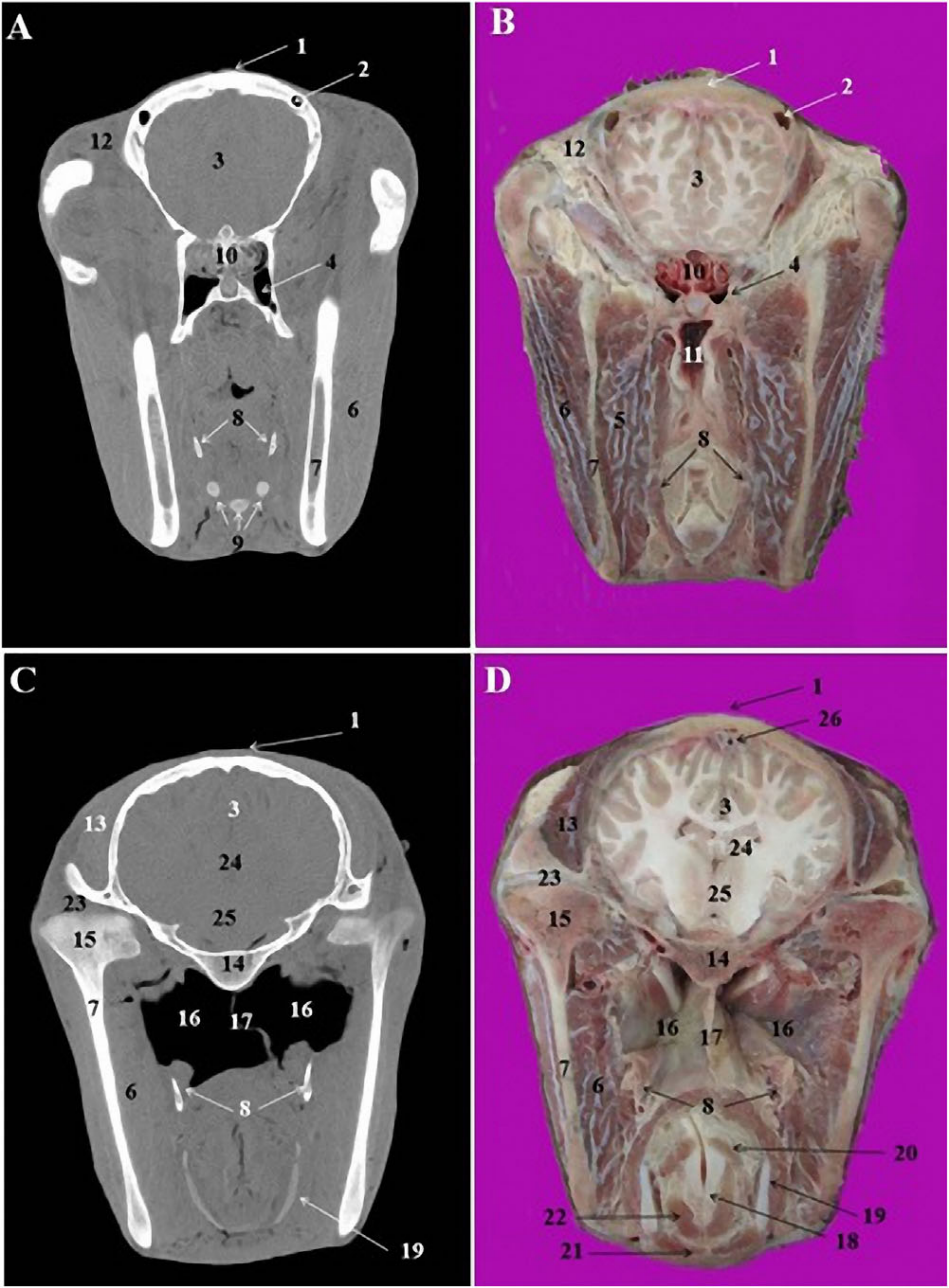
CT scan (a, c) and cross‐sectional (b, d) images of the middle nasal cavity at the level of the brain in the Arabian horse. 1: Frontal bone, 2: most caudal part of the frontal sinus, 3: frontal lobe of brain, 4: sphenopalatine sinus, 5: medial pterygoid muscle, 6: masseter muscle, 7: ramus of the mandible, 8: stylohyoid bones, 9: laryngeal muscles, 10: ethmoidal labyrinth, 11: pharyngeal recess, 12: retro‐orbital fat, 13: temporalis muscle, 14: body of basisphenoid bone, 15: condylar process of the mandible, 16: guttural pouch, 17: septum of guttural pouch, 18: arytenoid cartilage, 19: thyroid cartilage, 20: arythenoideous transversous, 21: cricothyroid muscle, 22: thyroarythrnoid muscle, 23: temporomandibular joint, 24: thalamus, 25: hypothalamus, 26: dorsal sagittal sinus of dura mater

**FIGURE 7:**
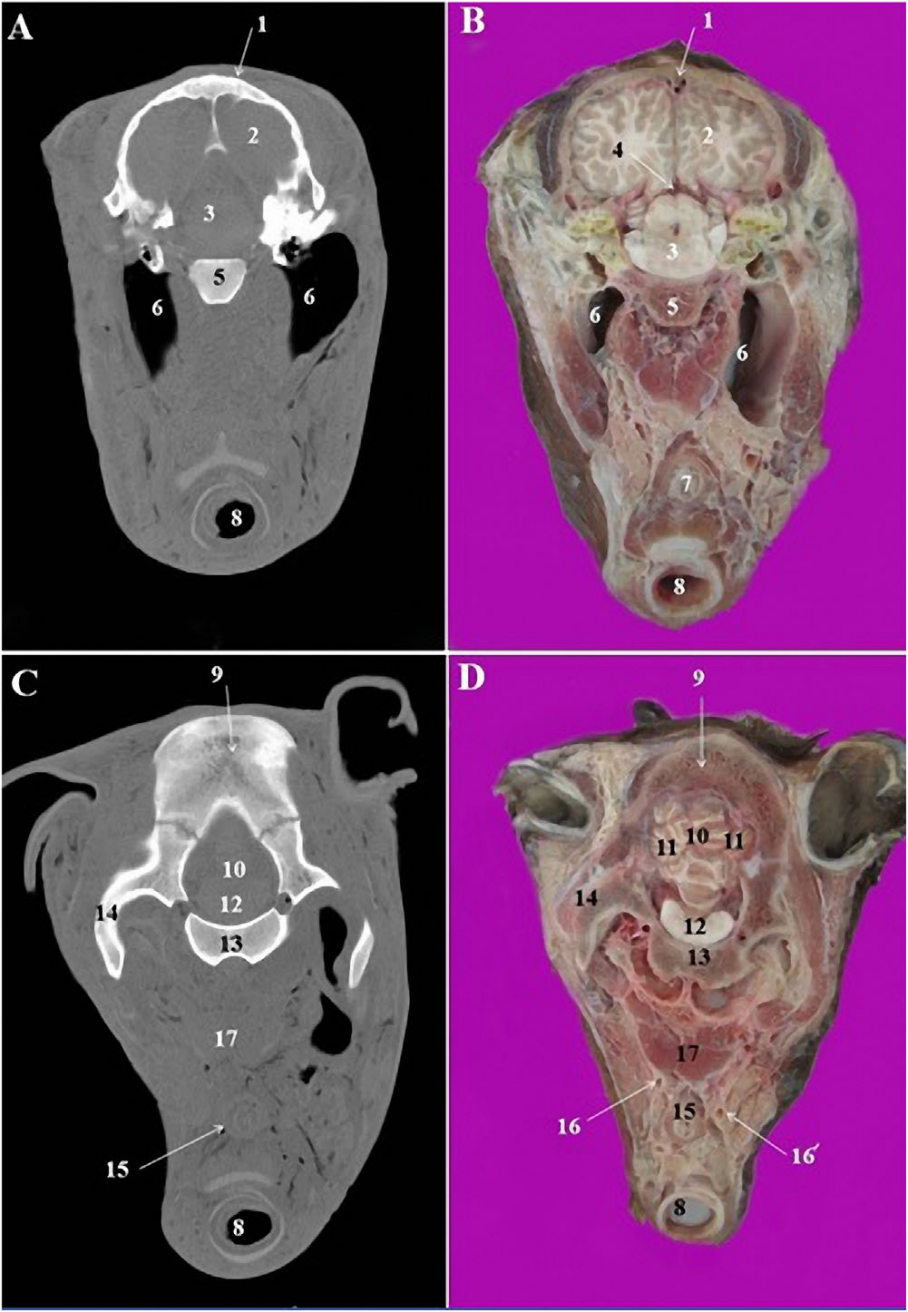
CT scan (a, c) and cross‐sectional (b, d) images of the cranial cavity at the level of the cerebellum in the Arabian horse. 1: Frontal bone, 2: parietal lobe of brain, 3: cerebral crura, 4: mesocephalic duct, 5: body of basisphenoid bone, 6: right and left guttural pouch, 7: oesophagus, 8: trachea, 9: parietal bone, 10: cerebellar vermis, 11: cerebellar hemisphere, 12: pons, 13: basilar part of occipital bone, 14: paracondylar process of occipital bone, 15: oesophagus, 16 and 16’: left and right common carotid artery, 17: longus capitis muscle

The bony structures and air‐filled cavities in the skull such as the nasal concha, nasal meatuses, conchal sinuses, paranasal sinuses, brain, eye and their appendices were easily recognized in the CT scan images (Figures 3, 4, 5, 6, 7).

The most relevant structures in the rostral nasal region of the nasal cavity were straight fold and alar fold (Figures 3/3, and 4/2, 6), while the dorsal, middle and ventral nasal concha and relevant nasal meatuses were identified in the CT scan images at the level of the middle and caudal nasal regions (Figures 3, 4/3, and 4/5).

The dorsal nasal concha was attached caudally to the cribriform plate of the ethmoid bone and contained the dorsal conchal sinus. This sinus was joined caudally to the frontal sinus and constitute the conchofrontal sinus (Figure 5/4). The later communicated with the nasal cavity through the caudal maxillary sinus. The middle nasal concha was seen on the caudal nasal region at the level of the 2nd lower molar teeth (Triadan 310 and 410) (Figure 5/15). The ventral nasal concha was divided into the dorsal and ventral scrolls at the level of the 3rd upper premolar (Triadan 107 and 207) and 2nd lower premolar teeth (Triadan 306 and 406) (Figure 4/18).

The cartilagous nasal septum was attached to the nasal bone dorsally and vomer bone's sulcus ventrally (Figures 3/2, 4/8, and 5/7). It was continued caudally with the perpendicular plate of the ethmoid bone.

Excluding conchal sinuses, the skull of the Arabian horse had three paranasal sinuses located into the bones of the cranial cavitiy: conchofrontal sinus, Maxillary sinus and sphenopalatine sinus.

The maxillary sinus was identified in the CT scan images at the level of the first upper molar tooth (Triadan 109 and 209). This sinus was divided by a complete bony septum into rostral and caudal compartments. The rostral maxillary sinus was contained the roots of the first ad second upper molar tooth (Triadan 109, 209, 110 and 210). Caudally, it continued as caudal maxillary sinus and encompassed the root of the third upper molar tooth (Triadan 111 and 211). The caudal maxillary sinus was connected to the middle nasal meatus via a slit‐like nasomaxillary aperture.

The frontal sinus was a small sinus which confined to the frontal bone. The rostral part of this sinus was integrated with the dorsal nasal concha and formed the conchofrontal sinus (Figure 5/4). Caudally, the frontal sinus was divided by few thin bony septum into the medial and caudal frontal sinuses (Figure 5/12, 13). The right and left frontal sinuses were separated with a thick and complete bony frontal septum (Figure 5/2). The sphenopalatine sinuses were very small sinuses in both palatine and sphenoid bones under the brain (Figure 6/4). This sinus communicates with the ethmoidal meatus via the sphenopalatinal opening.

The lens of the eye had a lower density in the obtained CT images (Figure 5/16, 18). The periorbital fat was also recognizable in the CT images (Figure 3/17). The CT and cross sections in the pharyngeal region identified nasopharynx, pharyngeal recess and guttural pouch (Figures 5/21, 6/11, 16). The right and left guttural pouches were separated by a thin mucosa, longus capitis and rectus capitis ventralis muscles (Figure 4/17). From the lateral aspect, the stylohyoid bone divided each guttural pouch into the medial and lateral compartments (Figure 4/8) which were extended caudally to the level of the parietal lobes of the brain (Figure 7/6).

The different parts of the brain and other appendices in the cranial cavity were accurately identified in the CT scan and cross‐anatomical sections. The frontal and parietal lobes of cerebrum were identified (Figures  6/3 and 7/2). The pons, cerebellar hemisphere and cerebellar vermis, thalamus and hypothalamus were also recognized (Figures 6/24, 25 and 7/10–12).

### 3.2 Volumetric study

The mean values estimated for paranasal sinuses volume in the Arabian horse are presented in Table 1. In this study, we focused on the main paranasal sinuses embedded in the skull bone; therefore, the conchal sinuses located in the nasal cavity were not included. The mean total volume of the rostral and caudal maxillary sinuses were estimated to be 131.93 ± 7.65 and 19.46 ± 2.15 cm^3^, respectively. The conchofrontal sinus occupied 116.82 ± 7.6 cm^3^ of the head volume and the sphenopalatine sinus had 13.3 ± 1.2 cm^3^ volume.

**TABLE 1:** Total volume (cm^3^) of the paranasal sinuses in the Arabian horse (*n* = 10)

No	Caudal maxillary sinus	Rostral maxillary sinus	Conchofrontal sinus	Sphenopalatine sinus
H1	131.41	22.81	112.25	13.25
H2	128.57	17.34	125.61	12.51
H3	147.53	21.52	109.63	10.98
H4	135.29	15.87	115.34	14.22
H5	127.94	19.55	131.57	12.83
H6	124.33	21.53	105.97	13.71
H7	131.82	19.65	113.84	13.51
H8	141.75	19.23	115.29	13.83
H9	126.71	17.38	117.22	15.53
H10	123.92	19.71	121.52	12.64
Mean	**131.93**	**19.46**	**116.82**	**13.3**
SD	**7.65**	**2.15**	**7.6**	**1.2**

### 3.3 Morphometric measurements

The obtained morphometric data are presented in the Table 2. The length of Bar was measured 4.5 ± 0.31 cm. The distance between the infraorbital foramen and most rostral point of facial crest was 4.16 ± 0.18 cm and the distance between the infraorbital canal and alveolar teeth was 4.70 ± 0.35 cm. The mental foramen was located on the bar region with a 3.12 ± 0.29 cm distance from the most lateral incisive tooth and 3.45 ± 0.22 cm distance from the first premolar tooth. The distance between the mental foramen and caudal border of the mandibular ramus and ventral border of mandibular body were 24.46 ± 0.85 cm and 1.94 ± 0.12 cm, respectively.

**TABLE 2:** Applied morphometric parameters (cm) of the Arabian horse skull (*n* = 10)

Parameters	Value
MFCB	24.46 ± 0.85
MFVB	1.94 ± 0.12
MFIT	3.12 ± 0.29
MFPT	3.45 ± 0.22
BL	4.51 ± 0.31
1F	4.16 ± 0.18
IA	4.70 ± 0.35
IP	5.79 ± 0.77

MFCB: mental foramen to the caudal border of the mandibular ramus, 2: MFVB: mental foramen to the ventral border of the mandibular body, MFIT: mental foramen to the most lateral incisive tooth, MFPT: mental foramen to the first premolar tooth, BL: bar length, IF: the distance between the infraorbital foramen and facial crest, IA: the distance between the infraorbital foramen to alvelolar tooth, IP: the distance between the infraorbital foramen and first upper premolar tooth.

## 4 DISCUSSION

In this study, an attempt has been made to provide some comprehensive and complementary information regarding the clinical anatomy of the skull, including the volumetric characteristics of the paranasal sinuses and the morphometric features of superficial nerves with reference to local anaesthesia. It is well known that the ability to identify the different anatomical structures in transverse or sagittal sections has an important role in the diagnosis of pathological conditions (Arencibia et al., 2000). In the CT scan images of the nasal cavity, due to the proximity of air and bony structures, nasal conchae, nasal meatuses and nasal septum as well as guttural pouch (Alsafy et al., 2008), eye (D'Août et al., 2015) and individual tooth roots are easily recognizable (Morrow et al., 2000).

In the present work three paranasal sinuses including conchofrontal, rostral and caudal maxillary, and sphenopalatine sinuses were identified in Arabian horse which were similar to those reported in previously examined horse breeds and donkey (Arencibia et al., 2000; El‐Gendy & Alsafy, 2010; El‐Gendy et al., 2014; Morrow et al., 2000; Nickel et al., 1986). Frontal sinus was triangular in shape united rostrally with the dorsal conchal sinus forming conchofrontal sinus, similar to those reported by Nickel et al. (1986) and Pobst et al. (2005) in horse.

In the present work, the maxillary sinus was divided by a bony septum into rostral and caudal maxillary sinuses which was in agreement with the previous reports (Pobst et al., 2005; Tremaine & Dixon, 2002). On the contrary, this bony septum was reported to be absent in the donkey (El‐Gendy & Alsafy, 2010; El‐Gendy et al., 2014). There is a considerable variation in the relationship between the maxillary sinuses and teeth roots. This relationship is usually affected by the forward migration of the teeth as they develop and come to wear. In the present study, it was observed that the roots of the 3rd maxillary molar teeth (Triadan 111 and 211) was embedded in the caudal maxillary sinus in 8 of 10 examined samples. This finding was in line with that stated by Amin and Kassem (1987) in the donkey. Also, Liuti et al. (2017) have shown that the Triadan 110 and 210 (2nd molar teeth) alveoli lay fully or partially in the rostral maxillary sinus in 60% cases. In newborn foal, only the last premolar (Triadan 108 and 208) and first molar teeth (Triadan 109 and 209) are embedded into the maxillary sinus and later it extends to involve the last four upper teeth (Dyce, 2018). It is known that the enlargement of the paranasal sinuses is continued up to 5 years old. It is noteworthy that additional to the sinuses, dorsal and ventral concha volume increase with animal age and their anatomical positions are closely associated with specific maxillay cheek teeth (Liuti et al., 2017).

Although the sphenopalatine sinus is the smallest paranasal sinus, it is considered to be of major clinical importance and could be affected by various disorders including empyema (Barnett et al., 2008), neoplasia (Bertuglia et al., 2006), fungal infection (Freeman, 1991) and progressive ethmoidal hematoma (Smith & Perkins, 2009). Moreover, due to the vicinity of this sinus with some vital structures such as optic nerve and maxillary branch of the trigeminal nerve, its pathological changes should be recognized accurately (Tucker et al., 2016). Therefore, knowledge about its anatomy is necessary for accurate interpretation of CT scan images.

In another part of this study, the normal values of paranasal sinuses volume were estimated using Cavalieri principle. The total volume of objects or cavities and fractional volume of their constituents can be easily estimated by this method (Simic et al., 1997). In human practice, this method can be coupled with transverse sections of the magnetic resonance imaging (MRI) and CT scan for estimating the normal volume or pathological volume changes of different organs such as brain and its ventricles (Ekinci et al., 2008; Sahin et al., 2003; Sullivan et al., 2002; Whitwell et al., 2007; Xenos et al., 2002) and liver (Sahin et al., 2003). Although volumetric measurement of the paranasal sinuses was performed previously in giraffe (Badlangana et al., 2011), Arabian foals (Bahar et al., 2014), rabbit (Ozkadif & Eken, 2013) and other rodent species (Philips et al., 2009), those studies, however, used non‐stereological methods. Also, in a recent study, Köhler et al. (2021) performed volumetric measurements of the paranasal sinuses in Sheltand ponies using of CT scan imaging. The combination of cross sections and stereological procedures has less frequently used for volume estimation of organs in veterinary medicine. Only a few studies were conducted on the spinal cord of sheep embryo (Sadeghinezhad et al., 2018), quail and ducks (Cakmak & Karadag, 2019; Cakmak & Ragbetli, 2019).

The volume of the conchofrontal sinus was estimated as 116.82 ± 7.6 cm^3^ in the adult Arabian horse. Whereas the volume of this sinus in the the Arabian foal was reported to be approximately 96 cm^3^ (Bahar et al., 2014), which was 21% lower than our result.

The volume of the rostral and caudal maxillary sinuses in the Arabian horse were 19.46 ± 2.15 and 131.93 ± 7.65 cm^3^, respectively. These values were estimated 6.7 and 22.8 cm^3^ in the Arabian foal (Bahar et al., 2014). This shows threefold and sixfold growth of the rostral and caudal maxillary sinuses with age. The volume of the sphenopalatine sinus was 13.3 ± 1.2 cm^3^ in the adult Arabian horses, which was slightly more than that described in the Arabian foal (11.4 cm^3^) (Bahar et al., 2014). The comparison of the sinuses volume between the Arabian foals and adults indicates most growth for caudal maxillary sinus and lowest development for sphenopalatine sinus with advancing age. Therefore, the caudal maxillary sinus and sphenopalatine sinus were the largest and smallest, respectively, in the adult Arabian horse. Due to the present findings and previous reports, it can be emphasised that the maxillary sinus, especially its caudal comparment undergoes most remarkable volumetric change toward adultness. However, more studies on larger populations of mature and young horses are necessary for improving these results. It is noteworthy that the above‐mentioned comparisons are made when the methods of estimating volumes were different.

Brinkschulte et al. (2013) used of semi‐automated segmentation of CT datasets for volume measurements of equine paranasal sinuses and reported that total volume of the paranasal sinuses and conchal sinuses in horse ranged from 911.5 to 1502 cm^3^. It should be considered, first, that in the present work, we focused on the paranasal sinuses of the cranil bones and, second, that a different method (design‐based stereology) was used in the present work. Therefore, the comparing the results should be done warily.

In equine, the facial crest is the most prominent anatomical feature which can be used as a superficial landmark to explore the infraorbital nerve. From a clinical point of view, detecting and blocking this nerve leads to anaesthetize the upper lip, nostril and skin of the face at the level of the infraorbital foramen. Therefore, the present data can be applied directly by veterinarians involved in practice of Arabian horse (Hall et al., 2000). The distance from facial crest to the infraorbital foramen (IF) was measured as 4.16 ± 0.18 cm in the Arabian horse, which was lower than that reported by Louei Monfared (2013) for Iranian horse (4.9 cm). It has been shown that infraorbital canal associated with nasomaxillary duct and frontomaxillary aperture is involved in sinusitis (Henninger et al., 2003). Furthermore, it has been demonstrated that various morphological changes in the infraorbital canal such as increased mineralization, decreased mineralization, deformed shape, displaced position and disruption can be associated with headshaking in horses with adjacent disease (Hermans & Veraa, 2019). Mental nerve is another clinically important cranial nerve which innervates the lower lip, lower gums, lower incisive teeth and the skin of the mental region. Therefore, knowing its exact location would be helpful in blocking this nerve. In this study, the mean distances from the mental foramen to the caudal border of the mandibular ramus (MFCB) and from the latter to the to the most lateral incisive tooth (MFIT) were measure 24.46 ± 0.85 and 3.12 ± 0.29 cm, respectively. These values were previously described in Iranian horse 21.1 ± 9.28 and 7.4 ± 0.33 cm. This comparison shows considerable differences for these values between the Arabian horse and Iranian horse breeds. In addition to these morphometric parameters, other anatomical criteria including foramen size, shape, width and height vary with gender and breed and must be considered with needle placement (Rawlinson et al., 2018).

In conclusion, the results of this study provided detailed information about the anatomy of the head in the Arabian horse. Due to the high economic value of this breed, the obtained data may be useful and applicable for enhancing its medical affairs and survival.

### PEER REVIEW

The peer review history for this article is available at https://publons.com/publon/10.1002/vms3.618

## ETHICAL STATEMENT

All procedures were conducted according to the guidelines for the animal welfare approved by the ethic committee of Razi University, Iran

## CONFLICT OF INTEREST

The authors have no conflict of interest.

## Data Availability

The data that support the findings of this study are available from the corresponding author upon reasonable request.
